# Novel design of weighted differential evolution for parameter estimation of Hammerstein-Wiener systems

**DOI:** 10.1016/j.jare.2022.02.010

**Published:** 2022-03-17

**Authors:** Ammara Mehmood, Muhammad Asif Zahoor Raja

**Affiliations:** a School of Electronics Engineering, Kyungpook National University, South Korea; b Future Technology Research Center, National Yunlin University of Science and Technology, 123 University Road, Section 3, Douliou, Yunlin 64002, Taiwan, ROC

**Keywords:** Hammerstein-Wiener system, Parameter estimation, Evolutionary heuristics, Weighted differential evolution, Genetic algorithms

## Abstract

**Introduction:**

Knacks of evolutionary computing paradigm-based heuristics has been exploited exhaustively for system modeling and parameter estimation of complex nonlinear systems due to their legacy of reliable convergence, accurate performance, simple conceptual design ease implementation ease and wider applicability.

**Objectives:**

The aim of the presented study is to investigate in evolutionary heuristics of weighted differential evolution (WDE) to estimate the parameters of Hammerstein-Wiener model (HWM) along with comparative evaluation from state-of-the-art counterparts. The objective function of the HWM for controlled autoregressive systems is efficaciously formulated by approximating error in mean square sense by computing difference between true and estimated parameters.

**Methods:**

The adjustable parameters of HWM are estimated through heuristics of WDE and genetic algorithms (GAs) for different degrees of freedom and noise levels for exhaustive, comprehensive, and robust analysis on multiple autonomous trials.

**Results:**

Comparison through sufficient large number of graphical and numerical illustrations of outcomes for single and multiple execution of WDE and GAs through different performance measuring metrics of precision, convergence and complexity proves the worth and value of the designed WDE algorithm. Statistical assessment studies further prove the efficacy of the proposed scheme.

**Conclusion:**

Extensive simulation based experimentations on measure of central tendency and variance authenticate the effectiveness of the designed methodology WDE as precise, efficient, stable, and robust computing platform for system identification of HWM for controlled autoregressive scenarios.

## Highlights

• A new application of WDE for estimation of nonlinear Hammerstein-Wiener systems.
• Designed WDE is implemented for measured input, internal and output data viably.
• The sundry scenarios of HWM establish robustness, stability and convergence of WDE.
• Statistical observations prove efficacy for controlled autoregressive scenarios.

## Graphical abstract

**Figure:**
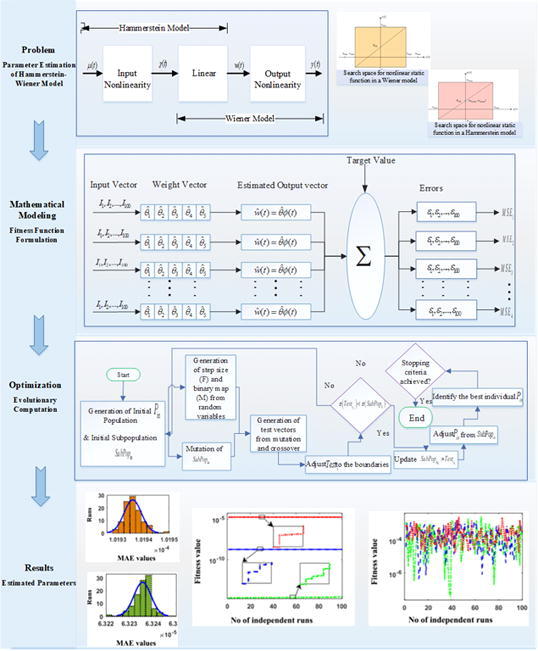

## Introduction

The block-structured models have emerged as a potential research topic in the domain of nonlinear parameter estimation, characterized by connections of linear dynamic and nonlinear static subsystems. Among the block-oriented structures, the Hammerstein and Wiener models are the most well-known, simple, and effective configurations with vast applications in chemical engineering, biomedical engineering, control systems, signal processing, and power electronics [1], [2], [3]. The Hammerstein model constitutes a static nonlinear block followed by linear dynamic block and the Wiener model comprises of a linear dynamic cascaded with a static nonlinearity [4]. The union of both the Hammerstein and the Wiener system generates the Hammerstein-Wiener model, which consists of one dynamic linear block sandwiched between two static nonlinear blocks having applications in all fields of science and engineering including nonlinear industrial processes [5], controls [6], signal processing [7], and instrumentation [8]. Several parameter estimation procedures have been formulated for identification of the Hammerstein-Wiener model, mainly including one-shot set-membership method [9], subspace method [10], blind approach [11], over parametrization [12], recursive least square algorithm [13], maximum likelihood method [14] iterative method [15] multi-signal-based method [16] and fractional approach [17]. All these proposed algorithms are deterministic procedures being broadly employed for the parameter estimation of Hammerstein-Wiener models having their own advantages, applications, and shortcomings but stochastic solvers based on evolutionary heuristics procedures have not yet been explored for the efficient parameter estimation of Hammerstein-Wiener models. Nature inspired heuristics based stochastic solvers have been extensively explored for constrained and unconstrained optimization problems arising in physical systems [18], [19], [20], [21] including plasma physics [22], astrophysics [23], atomic physics [24], electrical power systems [25], electrical machines [26], quantum mechanics [27], electronic devices [28], signal processing [29], electric circuits [30], nanofluidic systems[31], energy [32], computer virus models [33], biomedical engineering [34], thermodynamics [35] supply chain management [36], scheduling problem [37], and finance [38]. Furthermore, the evolutionary algorithms have also been applied for parameter estimation of block-oriented models including controlled autoregressive [39], controlled autoregressive moving average systems [40], Hammerstein [41], [42], wiener [43] and feedback nonlinear systems [44]. These applications motivated authors to exploit evolutionary computing heuristics as an alternate, accurate, and reliable parameter estimation technique for Hammerstein-wiener systems and their fractional variants developed on similar pattern as reported in [45], [46]. The potential features of the designed scheme are listed below:

• A new application of evolutionary heuristic paradigms based on Weighted Differential Evolution is introduced for accurate parameter estimation of nonlinear Hammerstein-Wiener systems and comparative analysis with counterparts to prove the worth and efficacy.
• The designed scheme is effectively implemented to estimate the model parameters in terms of measured input and output data, as well as, the internal variables associated with the prior estimations of the subsequent measures.
• The robustness, stability and convergence of the designed metaheuristic paradigm are established through decision variables of the Hammerstein-Wiener systems of different lengths corrupted with process noise scenarios.
• Statistical observations on measure on central tendency and variance further prove the efficacy of the designed methodology WDE as precise, efficient, stable, robust and alternate computing platform for system identification of HWM for controlled autoregressive scenarios.

Remaining of the article is structured as follows: In Section 2, the parameter estimation model of Hammerstein-wiener systems, problem formulation, explanation of designed methodology is given. In Section 3 the different models of Hammerstein -wiener system and their simulation results are provided in detail. While last section summarizes the study including conclusion and future recommendations.

## Design methodology

In this section, two-stage method is employed to estimate the parameters of Hammerstein-Wiener system, in the first phase, the mathematical models of the Hammerstein-Wiener systems are developed along with the cost function definition. In the second phase, evolutionary computing algorithms as optimization techniques are described to estimate the input as well as output nonlinear blocks parameters and noise model parameters of the Hammerstein-Wiener systems.

### Hammerstein-Wiener system model

**Fig. 1:**
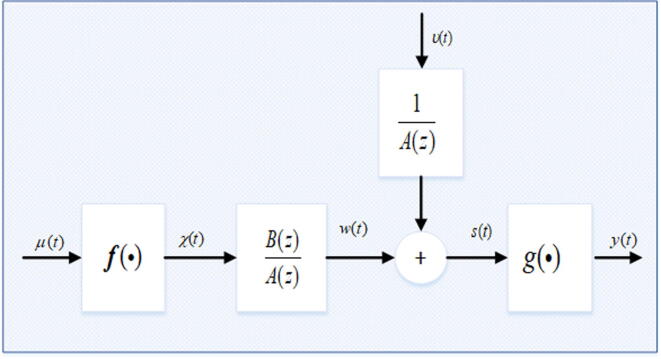
The generic block diagram of Hammerstein-Weiner system.

The Hammerstein-Wiener model is block-oriented model as shown in Fig. 1 is expressed mathematically as [30]:

(1) $$\chi \left(t\right)=f\left[\mu (t)\right],$$

(2) $$w\left(t\right)=\frac{B(z)}{A(z)}\chi \left(t\right),$$

(3) s(t)=w(t)+υ(t),

(4) $$y\left(t\right)=g\left(s(t)\right).$$

Where the system input is μ(t), y(t) is system’s output and υ(t) is stochastic white noise with zero mean, while w(t) and s(t) represents system internal variables. A(z), and B(z) are polynomials of known orders a and b, in the unit backward shift operator $z^{-1}$ and defined as:

(5) $$\left[\begin{array}{c} A(z)\\ B(z) \end{array}\right]=\left[\begin{array}{c} 1+\alpha_{1}z^{-1}+\alpha_{2}z^{-2}+...+\alpha_{a}z^{-a}\\ \beta_{1}z^{-1}+\beta_{2}z^{-2}+...+\beta_{b}z^{-b} \end{array}\right]$$

Moreover, χ(t) is the nonlinear block’s output having m basis functions $\left(f_{1},f_{2},...,f_{m}\right)$ with coefficients $\left(\lambda_{1},\lambda_{2},...,\lambda_{m}\right)$ as:

(6) $$\begin{array}{c} \chi (t)=f(\mu (t))\\ \;\;\;\;\;\;\;\;\;\;=\lambda_{1}f_{1}\left(\mu \left(t\right)\right)+\lambda_{2}f_{2}\left(\mu \left(t\right)\right)+...+\lambda_{m}f_{m}\left(\mu \left(t\right)\right) \end{array}$$

The output nonlinearity g is invertible and defined having basis functions $\left(g_{1},g_{2},...,g_{m}\right)$ with coefficients $\left(\gamma_{1},\gamma_{2},...,\gamma_{m}\right)$ as:

(7) $$\begin{array}{c} y(t)=g(s(t))\\ \;\;\;\;\;\;\;\;\;\;=\gamma_{1}g_{1}\left(s\left(t\right)\right)+\gamma_{2}g_{2}\left(s\left(t\right)\right)+...+\gamma_{n}g_{n}\left(s\left(t\right)\right) \end{array}$$

Using (1), (2), (5) and (6), χ(t) can be written as.

(8) $$\begin{array}{c} w(t)=[1-A(z)]w(t)+B(z)\chi (t)\\ \;\;\;\;\;\;\;\;\;\;=-\sum_{i=1}^{p}\alpha_{i}w\left(t-i\right)+\sum_{j=1}^{q}\beta_{j}\sum_{k=1}^{m}\lambda_{k}f_{k}\left[\mu \left(t-j\right)\right], \end{array}$$

Using (3) and (8), s(t) can be defined as:

(9) $$\begin{array}{c} s\left(t\right)=-\sum_{i=1}^{p}\alpha_{i}w\left(t-i\right)+\sum_{j=1}^{q}\beta_{j}\\ \;\;\;\;\;\;\;\;\;\;\;\;\;\;\;\;\;\;\;\;\;\;\;\;\;\;\;\;\;\;\;\;\;\;\;\;\;\;\;\;\;\;+\sum_{k=1}^{m}\lambda_{k}f_{k}\left[\mu \left(t-j\right)\right]+\upsilon \left(t\right), \end{array}$$

Substituting (9) in (7), we have:

(10) $$\begin{array}{c} g_{1}\left[s\left(t\right)\right]=-\sum_{l=2}^{n}\gamma_{l}g_{l}\left[s\left(t\right)\right]-\sum_{i=1}^{p}\alpha_{i}w\left(t-i\right)+\\ \;\;\;\;\;\;\;\;\;\;\;\;\;\;\;\;\;\;\;\sum_{j=1}^{q}\beta_{j}\sum_{k=1}^{m}\lambda_{k}f_{k}\left[\mu \left(t-j\right)\right]+\upsilon \left(t\right), \end{array}$$

The parameter vector for the Hammerstein-Wiener system from (10) is given as:

(11) $$\alpha :=\left[\begin{array}{c} \alpha_{1}\\ \alpha_{2}\\ \vdots \\ \alpha_{p} \end{array}\right]\in R^{p},\;\;\;\;\beta :=\left[\begin{array}{c} \beta_{1}\\ \beta_{2}\\ \vdots \\ \beta_{q} \end{array}\right]\in R^{q},\;\;\;\;\lambda :=\left[\begin{array}{c} \lambda_{1}\\ \lambda_{2}\\ \vdots \\ \lambda_{m} \end{array}\right]\in R^{m},\;\;\;$$

$$\begin{array}{c} \;\;\gamma :=\left[\begin{array}{c} \gamma_{1}\\ \gamma_{2}\\ \vdots \\ \gamma_{p} \end{array}\right]\in R^{n-1},\;\;\;\theta =\left[\begin{array}{c} \theta_{\alpha \gamma }\\ \theta_{\beta \lambda }\\ \theta_{\gamma } \end{array}\right]\in R^{n},\;\;\;\\ \;\left(n=pn+qm+n-1\right) \end{array}$$

$$\theta_{\alpha \gamma }=\left[\begin{array}{c} \gamma_{1}\alpha \\ \gamma_{1}\alpha \\ \vdots \\ \gamma_{1}\alpha \end{array}\right]\in R^{\mathrm{pn}},\;\;\;\theta_{\beta \lambda }=\left[\begin{array}{c} \lambda_{1}\beta \\ \lambda_{1}\beta \\ \vdots \\ \lambda_{1}\beta \end{array}\right]\in R^{\mathrm{qm}},\;\;\;\theta_{n}=\left[\begin{array}{c} \gamma_{2}\\ \gamma_{3}\\ \vdots \\ \gamma_{n} \end{array}\right]\in R^{n-1}$$

$$\psi_{g}\left(t\right):=\left[\begin{array}{c} g_{2}\left[s\left(t\right)\right]\\ g_{3}\left[s\left(t\right)\right]\\ \vdots \\ g_{n}\left[s\left(t\right)\right] \end{array}\right]\in R^{n-1},\;\;\psi_{w}\left(t\right):=\left[\begin{array}{c} w(t-1)\\ w(t-2)\\ \vdots \\ w(t-p) \end{array}\right]\in R^{p},\;\;\;$$

$$\begin{array}{c} \psi_{\mu }\left(t\right):=\left[\begin{array}{cccc} f_{1}\left[\mu \left(t-1\right)\right] & f_{2}\left[\mu \left(t-1\right)\right] & \cdots & f_{m}\left[\mu \left(t-1\right)\right]\\ f_{1}\left[\mu \left(t-2\right)\right] & f_{2}\left[\mu \left(t-2\right)\right] & \cdots & f_{m}\left[\mu \left(t-2\right)\right]\\ \vdots & \vdots & \vdots \\ f_{1}\left[\mu \left(t-q\right)\right] & f_{2}\left[\mu \left(t-q\right)\right] & \cdots & f_{m}\left[\mu \left(t-q\right)\right] \end{array}\right]\in R^{qxm},\\ \psi \left(t\right):=\left[\begin{array}{c} \begin{array}{c} \psi_{y}\left(t\right)\\ \psi_{\mu }\left(t\right) \end{array}\\ \psi_{w}\left(t\right) \end{array}\right]\in R^{n}, \end{array}$$

Using (11) gives:

(12) $$y\left(t\right)\;=-\psi^{T}\left(t\right)\theta +\upsilon \left(t\right).$$

The Thiel's inequality coefficient index is employed for the error function formulation of Hammerstein-Wiener models as:

(13) $$\varepsilon =\sqrt{\frac{1}{l}\sum_{i=1}^{l}{\left(y_{i}-{\hat{y}}_{i}\right)}^{2}}/\left(\sqrt{\frac{1}{l}\sum_{i=1}^{l}y_{i}^{2}}+\sqrt{\frac{1}{l}\sum_{i=1}^{l}{\hat{y}}_{i}^{2}}\right)$$

here $y(t_{i})$ stands for desired output for *i*^th^ observation and $\hat{y}\left(t_{i}\right)$ for estimated response of the actual output, while *l* represents the total number of instances. The estimated response $y(t_{l})$ for with respective information vector $\psi^{T}\left(t_{l}\right)$ is mathematically written as:

(14) $$\hat{y}\left(t_{l}\right)=\left(\psi^{T}\left(t_{l}\right)\hat{\theta }\right),$$

(15) $$\varepsilon =\frac{1}{I}\sum_{l=1}^{I}{\left(\left(\psi^{T}\left(t_{l}\right)\theta +v\left(t_{l}\right)\right)-\left(\psi^{T}\left(t_{l}\right)\hat{\theta }\right)\right)}^{2}$$

For the ideal case of parameter estimation, the estimated output $\;\hat{y}$ approach its optimal y as ε→0.

### Optimization procedure

Metaheuristic evolutionary strategies like WDE and GAs that are proposed in this work for parameter estimation of Hammerstein-Wiener model are briefly explained here.

WDE is a latest bi-population algorithm from the family of evolutionary heuristics developed to solve nearly all real-valued unimodal and multimodal optimization problems [47]. WDE is capable of efficiently finding evolutionary search direction. Additionally, in WDE population diversity remain stable and does not decline swiftly which leads to effective searches in next upcoming iterations. A few benchmark problems include GPS network adjustment problem, Pressure-vessel, Speed-reducer Welded-beam design [47], and camera calibration [48].

GAs introduced by Holland [49], is a stochastic, effective, and broadly used evolutionary computation algorithm developed to solve real-valued numerical optimization problems [49]. GAs is easy implemented, robust, simple, efficient and reliable global search algorithm that uses three basic operators, like crossover, mutation and selection for generating new efficient population with better fitness [50]. The individuals with better fitness are less likely to trap in local minima. Few recent applications of GAs include electrical circuits [50], supply chain [51], lungs cancer [52] and wind speed prediction [53].

**Fig. 2:**
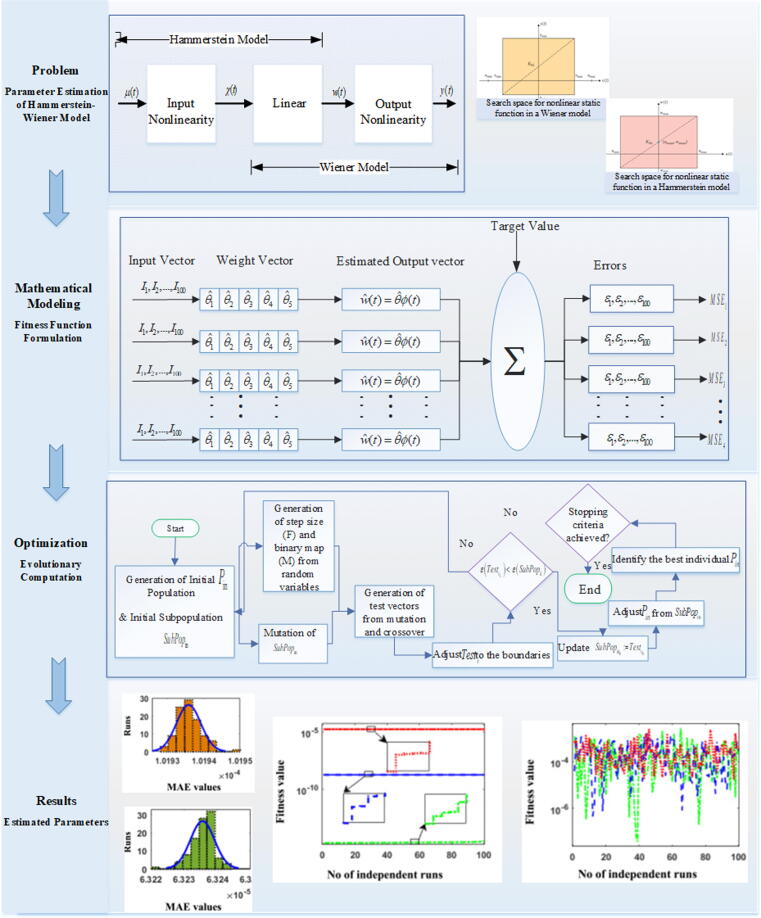
Workflow Diagram of parameter estimation problem of nonlinear Wiener system.

**Fig. 3:**
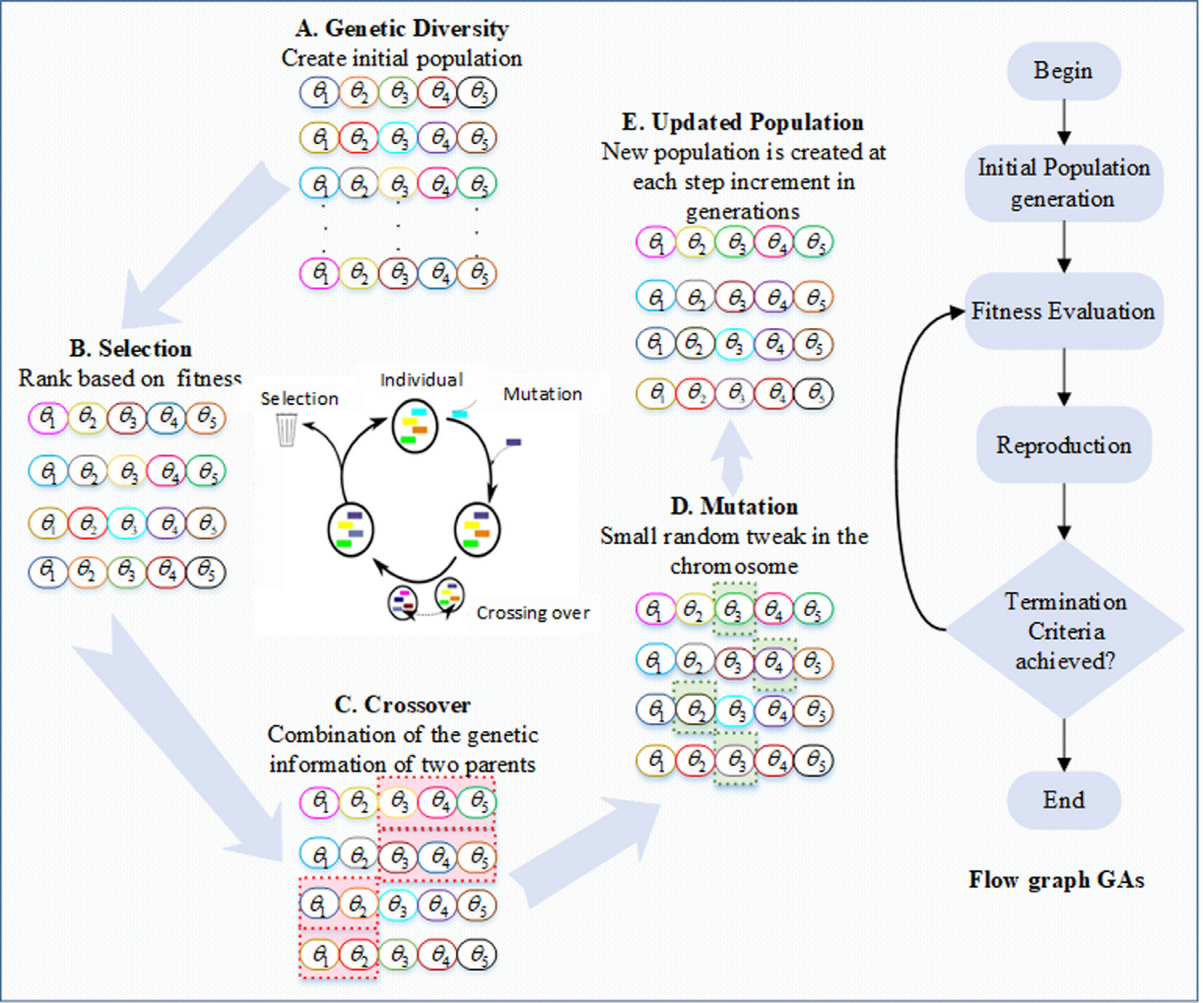
The generic block diagram of Hammerstein.

Efficacy of WDE, and GAs are the inspirations to the authors to use these evolutionary heuristics for finding optimal parameters of Hammerstein-Wiener system. Flow chart with procedural steps of GAs is shown in Fig. 3. Furthermore, the detailed stepwise procedures of WDE for Hammerstein-Wiener model is given as pseudocode form in Table 1. The designed methodology of the proposed work is illustrated in Fig. 2.

**Table 1:** Pseudocode of WDE.

### Performance indices

In this study, four performance operators i.e.*,* $\mathrm{MA}E_{hw\theta }$, $\mathrm{MW}D_{hw\theta }$, $\mathrm{RMS}E_{hw\theta }$, and $\mathrm{TI}C_{hw\theta }$ are utilized to validate the performance of the proposed evolutionary algorithms. These performance operators along with their mathematical description with respect to true and estimated parameters are provided in this section.

$\mathrm{MA}E_{hw\theta }$ is mathematically written as:

(16) $$\mathrm{MA}E_{hw\theta }=\frac{1}{W}\sum_{\mathrm{hw}=1}^{W}\left|\theta_{\mathrm{hw}}-{\hat{\theta }}_{\mathrm{hw}}\right|,$$

$\mathrm{MW}D_{hw\theta }$ is mathematically written as:

(17) $$\mathrm{MW}D_{hw\theta }=\frac{∥{\hat{\theta }}_{\mathrm{hw}}-\theta_{\mathrm{hw}}∥}{∥\theta_{\mathrm{hw}}∥},$$

$\mathrm{RMS}E_{hw\theta }$ is defined as:

(18) $$\mathrm{RMS}E_{hw\theta }=\sqrt{\frac{1}{W}\sum_{\mathrm{hw}=1}^{W}{\left(\theta_{\mathrm{hw}}-{\hat{\theta }}_{\mathrm{hw}}\right)}^{2},}$$

$\mathrm{TI}C_{hw\theta }$ mathematical formulation is as follows:

(19) $$\mathrm{TI}C_{hw\theta }=\frac{\sqrt{\frac{1}{W}\sum_{\mathrm{hw}=1}^{W}{\left(\theta_{\mathrm{hw}}-{\hat{\theta }}_{\mathrm{hw}}\right)}^{2}}}{\left(\sqrt{\frac{1}{W}\sum_{\mathrm{hw}=1}^{W}\theta_{\mathrm{hw}}^{2}}+\sqrt{\frac{1}{W}\sum_{\mathrm{hw}=1}^{W}{\hat{\theta }}_{\mathrm{hw}}^{2}}\right)}.$$

The magnitude of these performance metrics should approach zero for an ideal model.

## Results and discussion

In this section, results of the experiments are discussed for two different examples of Hammerstein-Wiener system on the basis of different length of parameter vector and noise variances conducted though evolutionary computing heuristics WDE and GAs.

### Model: I

In this Hammerstein-Wiener model, five unknown entities in the parameter vector are taken for estimation with polynomial type nonlinearity in both input and output typt nonlinearity and is mathematical form as:

$$w\left(t\right)=\frac{B(z)}{A(z)}f\left[\mu (t)\right],$$

s(t)=w(t)+υ(t),

(19) $$y\left(t\right)=g\left(s(t)\right).$$

$$A\left(z\right)=1+0.55z^{-1},$$

$$B\left(z\right)=0.15z^{-1},$$

$$\chi \left(t\right)=0.5\mu \left(t\right)+0.18\mu^{2}\left(t\right),$$

$$y\left(t\right)=s\left(t\right)+0.15s^{2}\left(t\right).$$

The objective function of nonlinear nonlinera Wiener model example 1 is formulated as described in equation (22) with K = 20 and N = 6 as:

(20) $$\varepsilon =\frac{1}{20}\sum_{l=1}^{20}{\left(\left(\psi^{T}\left(t_{l}\right)\theta +v\left(t_{l}\right)\right)-\left(\psi^{T}\left(t_{l}\right)\hat{\theta }\right)\right)}^{2}$$

### Model: II

In this example, eight unknown parameters are taken for estimation of nonlinear Hammerstein Wiener model with polynomial type output nonlinearity and is mathematically defined as:

$$w\left(t\right)=\frac{B(z)}{A(z)}f\left[\mu (t)\right],$$

s(t)=w(t)+υ(t),

(21) $$y\left(t\right)=g\left(s(t)\right).$$

$$A\left(z\right)=1+0.55z^{-1}+0.80z^{-2},$$

$$B\left(z\right)=0.15z^{-1}-0.35z^{-2},$$

$$\chi \left(t\right)=0.5\mu \left(t\right)+0.18\mu^{2}\left(t\right)-0.15\mu^{3}\left(t\right),$$

$$y\left(t\right)=s\left(t\right)+0.15s^{2}\left(t\right).$$

Likewise, the objective function of nonlinear nonlinear Wiener model example 2 is given as:

(22) $$\varepsilon =\frac{1}{20}\sum_{l=1}^{20}{\left(\left(\psi^{T}\left(t_{l}\right)\theta +v\left(t_{l}\right)\right)-\left(\psi^{T}\left(t_{l}\right)\hat{\theta }\right)\right)}^{2}$$

**Fig. 4:**
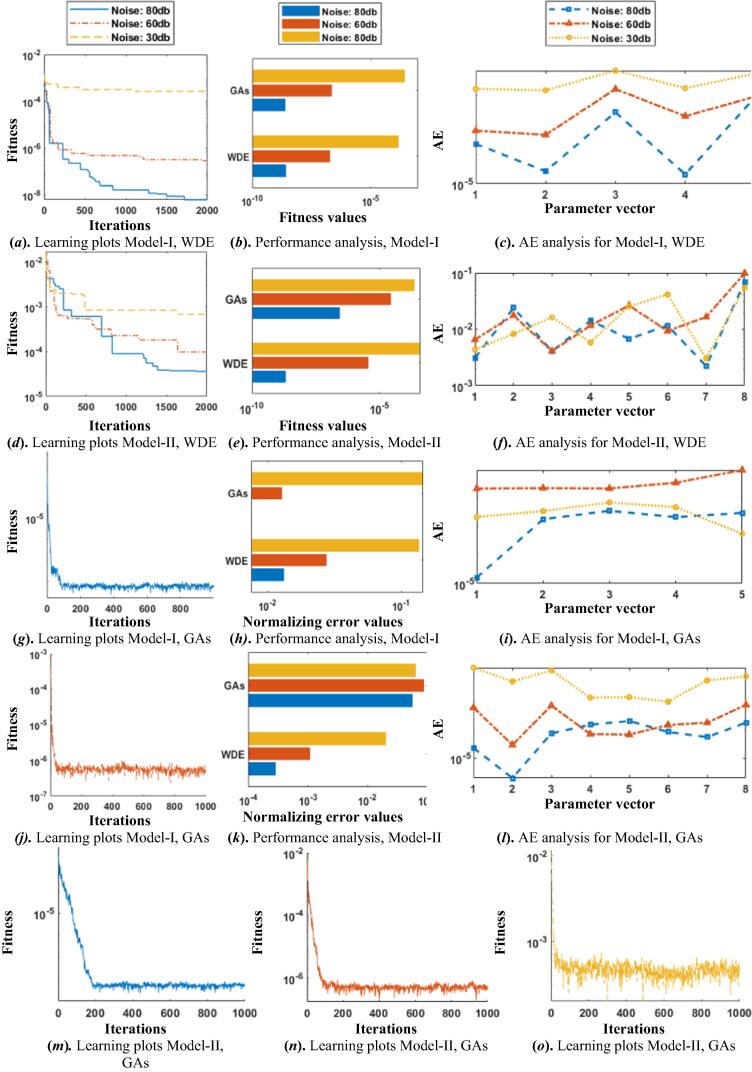
Plots for Iterative adaptation of fitness function for the HW model.

**Fig. 5:**
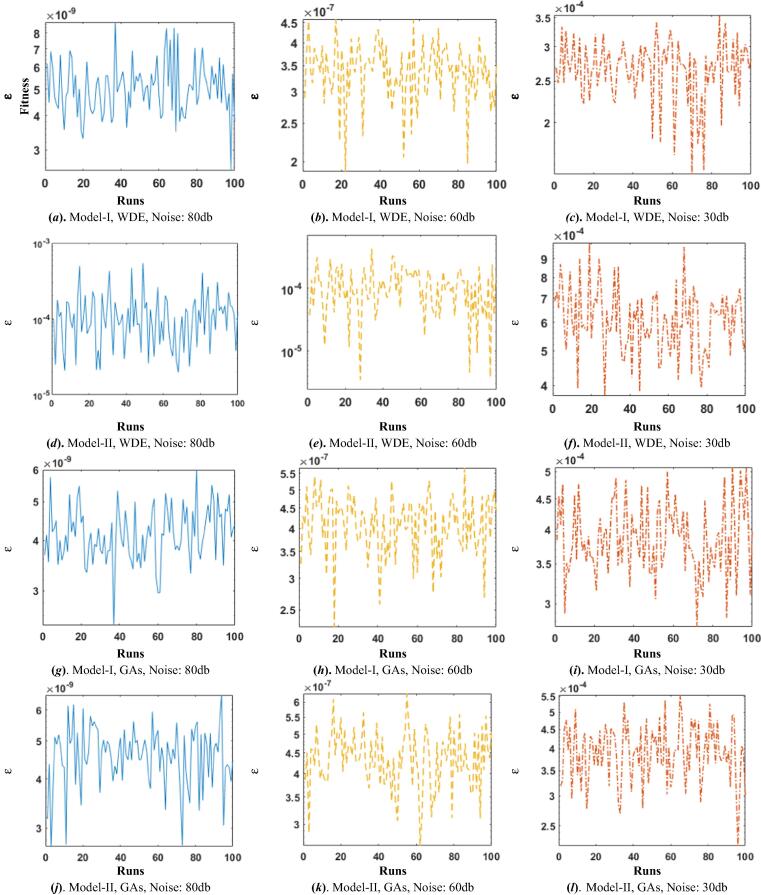
Plots based on fitness values for the HW model.

In these two Hammerstein Wiener models, input signal is taken as randomly generated signal of zero mean and unit variance, while noise is also a random signal with mean zero and constant variance. The parameter estimation of Hammerstein Wiener models is performed through the wellknown evolutionary computational heuristics i.e., WDE and GAs for optimization of fitness functions for 20 snap shots. The results of proposed scheme based on learning curves along with absolute error analysis are given in Fig. 4. The iterative convergence graphs of fitness in case of WDE for Model-I and II are presented in subfigs. 4(a) and (d), respectively while for GAs the learning curves are shown in subfig. 4(g) for Model-I, 80db noise, subfig. 4(j) Model-I, 60db noise, subfigs. 4(m-o) and Model-II, 80db, 60db and 30db noise levels, respectively. It is observed that both the algorithms are convergent but convergence of WDE is slightly superior than GAs. Comparison based on fitness values are also shown here in subfig 5(b) and 5(e) for Model I and II, respectively, while normalizing error plots comparisons are presented in subfig 5(h) and 5(k) for Model I and II respectively. In these plots, it can be observed that fitness values achieved by WDE is higher than GAs, also it can be seen that fitness valus decreases as the noise level increases. Along with this comparison, absolute errors (AEs) for the two Hammerstein-Wiener model for the three noise scenarios are shown in graphical form in subfig 5(f) and 5(i) for Model I and II respectively in case of WDE and in subfig 5(c) and 5(f) for Model I and II respectively in case of WDE while in subfig 5(i) and 5(l) for Model I and II respectively in case of GAs. The AE magnitudes are found in the range of 10^−9^, 10^−7^, 10^−4^ for noise levels 80db, 60db and 30db with WDE, and 10^−7^, 10^−5^, 10^−4^ for noise levels 80db, 60db and 30db with GAs for Model-I. while almost similar trend is found for GAs. Consistent accuracy is found for both the algorithms while slight degradration in the accuracy is observed with an increase in the noise levels for the proposed schemes.Fig. 5.

**Fig. 6:**
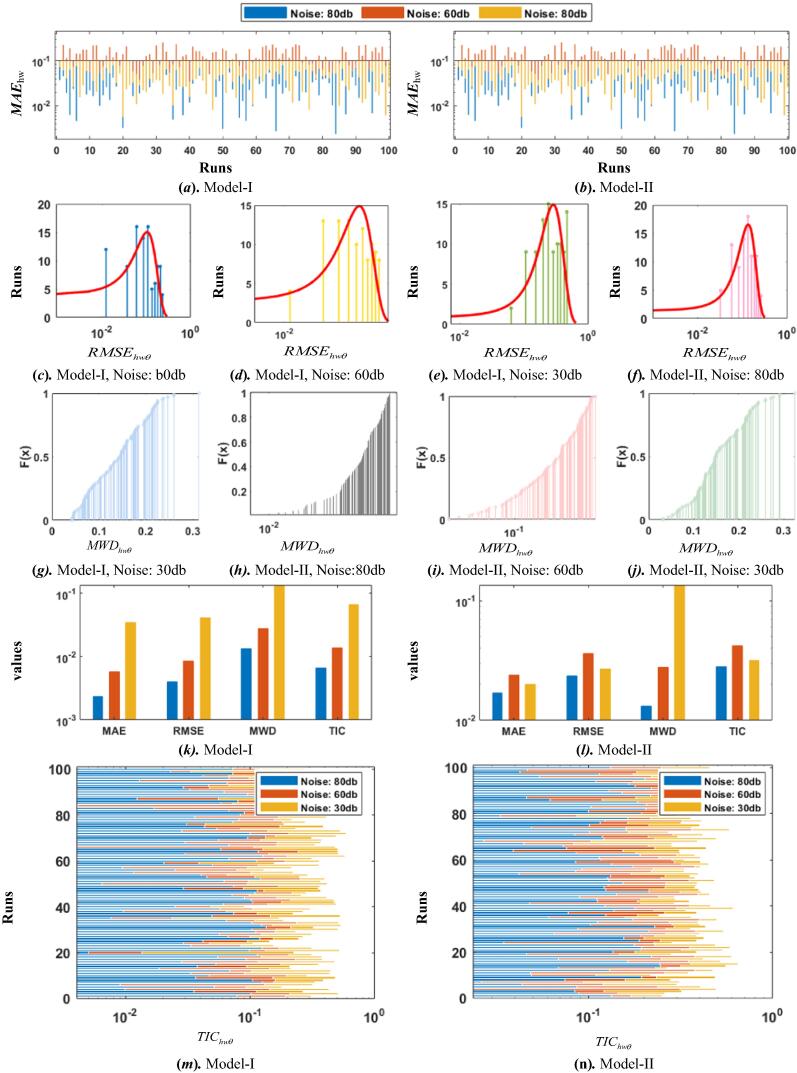
Comparison of the results based on performance indices for HW model for WDE.

Analysis of accuracy of the designed scheme is performed for 100 iterations for the two models of Hammerstein-Wiener system and results based on fitness values are plotted in semilogrithmic style for better analysis in Fig. 6. It can be observed that the respective magnitudes found close to 10^−9^, 10^−7^, 10^−4^ with noise levels 80db, 60db and 30db in case of Model-I, and 10^−5^, 10^−4^, 10^−3^ with noise levels 80db, 60db and 30db for in case of Model-II with WDE.Almost similar trend is found for GAs. Very small high fitness values proves the accuracy of the scheme.

The efficacy and relaiblility of the proposed evolutionary heuristics is validated through performance indices *i.e*., error function, $\mathrm{MW}D_{hw\theta }$, $\mathrm{MA}E_{hw\theta }$, $\mathrm{RMS}E_{hw\theta }$, and $\mathrm{TI}C_{hw\theta }$. Results in term of the best run based on minimum fitness, as well as the complexity measures based on time, generations consumed, and function counts are listed in Table 2. It is quite clear that the $\mathrm{MW}D_{hw\theta }$ noticed close to 10^−3^ and 10^−2^ for Model I with WDE and GAs, while in case of Model II, magnitudes are found close to 10^−2^ and 10^−1^, for Model II with WDE and GAs. The more values close to zeros of the performance indices proves the consistency and precision of the proposed evolutionary algorithms.

**Table 2:** Performance comparison on the best runs of WDE and GAs algorithm.

**Scheme**	**Algorithm**	**Noise db**	**Accuracy Measures**					**Complexity Measures**		
			***e***	$\mathrm{MW}D_{hw\theta }$	$\mathrm{MA}E_{hw\theta }$	$\mathrm{RMS}E_{hw\theta }$	$\mathrm{TI}C_{hw\theta }$	**Time**	**Gens**	**Funcount**
**WDE**	**I**	**80**	5.65E−09	1.32E−02	2.35E−03	4.05E−03	6.61E−03	1.55	2000	52,052
		**60**	3.84E−07	1.64E−02	5.71E−03	8.44E−03	1.38E−02	1.62	2000	52,052
		**30**	2.88E−04	8.19E−02	3.46E−02	4.14E−02	6.60E−02	1.47	2000	52,052
	**II**	**80**	1.98E−05	3.24E−02	1.69E−02	2.36E−02	2.81E−02	1.55	2000	52,052
		**60**	3.64E−05	2.18E−01	2.38E−02	3.60E−02	4.22E−02	0.77	2000	52,052
		**30**	5.60E−04	3.09E−02	1.99E−02	2.68E−02	3.17E−02	0.47	2000	52,052
**GAs**	**I**	**80**	4.80E−09	7.55E−03	2.01E−03	2.31E−03	3.77E−03	1000	1000	320,320
		**60**	3.86E−07	4.62E−01	3.38E−03	2.31E−03	6.44E−03	1000	1000	320,320
		**30**	3.58E−04	9.74E−01	3.61E−02	2.31E−03	7.37E−02	1000	1000	320,320
	**II**	**80**	4.78E−09	5.22E−01	9.90E−05	2.31E−03	1.45E−04	142.09	1000	320,320
		**60**	4.08E−07	3.86E−01	3.33E−04	2.31E−03	5.37E−04	137.56	1000	320,320
		**30**	4.58E−04	2.40E−01	6.59E−03	2.31E−03	1.01E−02	131.62	1000	320,320

**Fig. 7:**
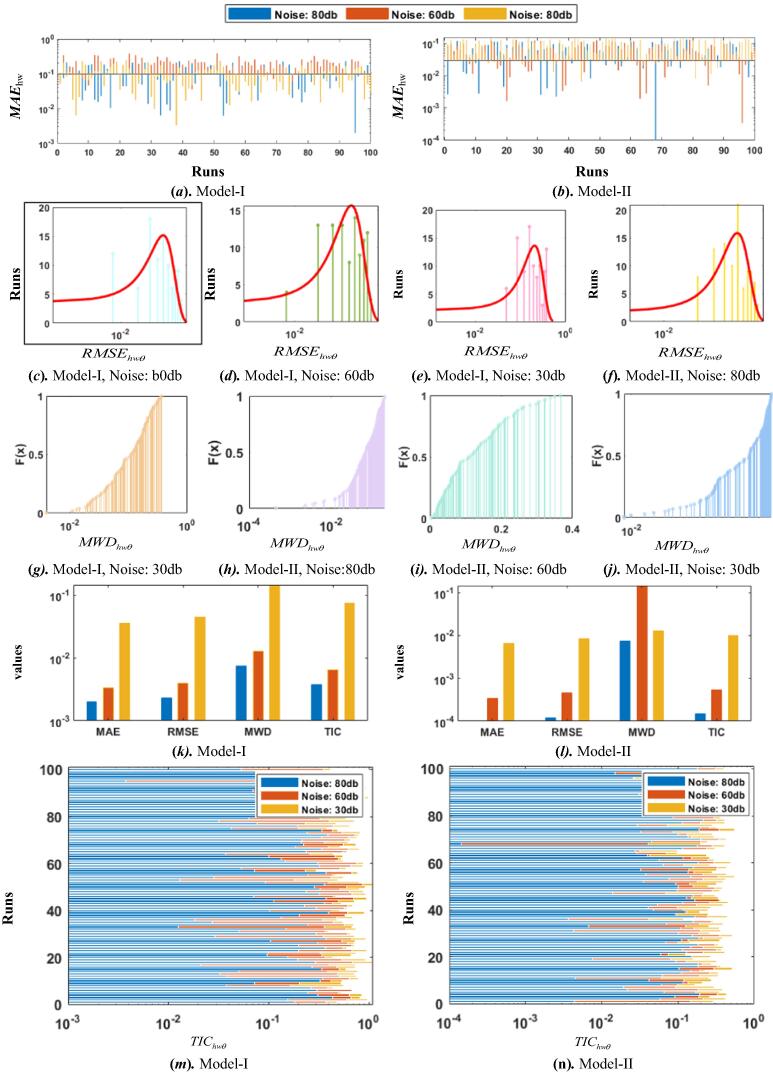
Comparison of the results based on performance indices for HW model for GAs.

Comparison via different performance indices i.e., error function, $\mathrm{MW}D_{hw\theta }$, $\mathrm{MA}E_{hw\theta }$, $\mathrm{RMS}E_{hw\theta },$ and $\mathrm{TI}C_{hw\theta }$ are employed to further examine the accuracy of the designed schemes and shown in pictoral form in Fig. 7 for WDE and GAs, respectively. The $\mathrm{MA}E_{hw\theta }$ magnitudes for both the models are shown in graphical form for 100 independent runs in subfig. 7(a), and subfig. 7 (b), for Model I and II, respectively. In case of WDE and in subfig. 8 (a), and subfig. 8 (b), for Model I and II, respectively. In case of GAs. It can be seen from the figures that the MAE values for WDE in case of Model I and II are found in the range of 10^−3^ to 10^−1^ for noise levels 80db, 60db and 30db. In order to further examine the precision, results as the plots of histogram are investigated based on RMSE values for both WDE and GAs are shown in subfigs. 6 (*c-f*), and subfigs. 7 (*c-f*), respectively. In addition to histogram graphs, empirical Cumulative Distribution Function graphs are also plotted in terms of MWD magnitudes for Hammerstein Wiener models with both designed schemes and are shown in subfigs. 6 (*g-j*), and subfigs. 7 (*g-j*), respectively, for WDE and GAs, respectively. In subfigs. 6 (*k-l*), and subfigs. 7 (*k-l*), comparative bar graphs are ploted both with WDE and GAs, respectively for Model I and II. In order to endorse the accuracy further, stacked bar graphical illustrations are also shown in terms of TIC values for Model I and II in subfigs. 6 (*m-n*), and subfigs. 7 (*m-n*), for WDE and GAs, respectively. and similar trend is found for GAs outcomes. These all graphical illustrations validate the consistent accuracy of the two proposed heuristic strategies for the parameter estimation of Hammerstein Wiener systems.

Further analysis of precision on 100 independent runs of the algorithms is carried out through the statistical performance measures via mean, best, and worst values of fitness and statistical outcomes for the two proposed evoltuionary heuristics are listed in Table 3, Table 4 for respective Models I and II with three noise level added in Hammerstein Wiener system. It can be observed that with the increase in the noise level, there is a decrease in the performance of the WDE and GAs, and same is the trend with the also with the increase in the unknown poarameters of the model as less degree of freedom make parameter estimation problem more stiff.

**Table 3:** Comparative analysis through results of statistics for Model II of system.

**Model**	**Algorithm**	**Noise** **(db)**	**Values**	**Parameter Vector**				
				***i* = 1**	***i* = 2**	***i* = 3**	***i* = 4**	***i* = 5**
**I**	**WDE**	**80**	**Best**	0.550	0.150	−0.303	0.180	0.141
			**Mean**	0.550	0.150	−0.234	0.180	0.151
			**Worst**	0.550	0.150	0.000	0.180	0.143
		**60**	**Best**	0.551	0.150	−0.284	0.182	0.159
			**Mean**	0.550	0.150	−0.242	0.179	0.177
			**Worst**	0.555	0.148	−0.390	0.181	0.470
		**30**	**Best**	0.532	0.165	−0.369	0.162	0.204
			**Mean**	0.522	0.144	−0.252	0.187	0.507
			**Worst**	0.646	0.113	−0.474	0.328	0.978
	**GA**	**80**	**Best**	0.550	0.148	−0.303	0.182	0.153
			**Mean**	0.550	0.126	−0.512	0.307	0.150
			**Worst**	0.550	0.047	−0.950	0.570	0.150
		**60**	**Best**	0.552	0.153	−0.294	0.176	0.149
			**Mean**	0.550	0.150	−0.458	0.275	0.151
			**Worst**	0.550	0.045	−0.993	0.604	0.131
		**30**	**Best**	0.530	0.129	−0.320	0.212	0.062
			**Mean**	0.551	0.084	−0.603	0.354	0.432
			**Worst**	0.552	0.054	−0.944	0.566	0.977
**Actural *θ***				**0.550**	**0.150**	**−0.3--**	**0.180**	**0.150**

**Table 4:** Comparison through results of statistics for Model II of system.

**Model**	**Algorithm**	**Noise** **(db)**	**Value**	**Parameter Vector**							
				***i* = 1**	***i* = 2**	***i* = 3**	***i* = 4**	***i* = 5**	***i* = 6**	***i* = 7**	***i* = 8**
**II**	**WDE**	**80**	**Best**	0.55	0.82	0.15	−0.36	0.49	0.17	−0.15	0.08
			**Mean**	0.54	0.78	0.11	−0.26	0.70	0.27	−0.20	0.27
			**Worst**	0.44	0.63	0.07	−0.17	1.00	0.41	−0.25	0.45
		**60**	**Best**	0.56	0.78	0.15	−0.34	0.53	0.19	−0.17	0.05
			**Mean**	0.54	0.79	0.11	−0.25	0.72	0.28	−0.21	0.24
			**Worst**	0.50	0.73	0.07	−0.16	1.00	0.50	−0.22	0.44
		**30**	**Best**	0.55	0.81	0.13	−0.34	0.53	0.22	−0.15	0.10
			**Mean**	0.54	0.77	0.11	−0.26	0.70	0.28	−0.19	0.32
			**Worst**	0.44	0.68	0.07	−0.21	0.98	0.40	−0.26	0.62
	**GAs**	**80**	**Best**	0.55	0.80	0.15	−0.35	0.50	0.18	−0.15	0.15
			**Mean**	0.55	0.80	0.12	−0.28	0.68	0.24	−0.20	0.15
			**Worst**	0.55	0.80	0.09	−0.22	0.81	0.29	−0.24	0.15
		**60**	**Best**	0.55	0.80	0.15	−0.22	0.80	0.29	−0.24	0.15
			**Mean**	0.55	0.80	0.12	−0.22	0.67	0.24	−0.20	0.15
			**Worst**	0.55	0.80	0.09	−0.28	0.77	0.28	−0.23	0.15
		**30**	**Best**	0.56	0.80	0.08	−0.20	0.91	0.30	−0.26	0.18
			**Mean**	0.55	0.80	0.11	−0.25	0.76	0.27	−0.23	0.15
			**Worst**	0.56	0.82	0.13	−0.29	0.57	0.20	−0.17	0.10
**Actural *θ***				**0.55**	**0.80**	**0.15**	**0.35**	**0.50**	**0.18**	**−0.15**	**0.15**

Complexity analysis is computed for the proposed evolutionary algorithms WDE and GAs via average time spent, mean generations executed and average times fitness functions are executed during optimization for finding optimal parameters of Hammerstein Wiener models. Computational complexity was analysed for 100 independent runs of both evolutionary schemes and are listed in Table. 5. It can be seen that the values of average time executed, generations completed and functions evaluation are around 0.35 ± 0.004, 200, 8040 for WDE, and 1.13 ± 0.096, 600, 24,040 for GAs, in case of Model I. It can be seen that among these two evolutionary heuristic algorithm, WDE is relatively less computationaly complex than that of GAs. Also with the increase in the dimensionality of the Hammerstein Wiener model, the computational complexity of WDE and GAs rises. All the computational work is performed on computer station, having Intel(R) Core (TM) i7-4770 CPU @3.40 GHz processor, 8 GB RAM.

**Table 5:** Comparison through complexity operators for system.

**Algorithm**	**Mode**	**Noise** **(db)**	**Complexity measures**					
			**Time**		**Generations**		**Function counts**	
			**Mean**	**STD**	**Mean**	**STD**	**Mean**	**STD**
**WDE**	**I**	80	0.358	0.003	200	0	8040	0
		60	0.362	0.004	200	0	8040	0
		30	0.358	0.002	200	0	8040	0
	**II**	80	0.699	0.004	400	0	16,040	0
		60	0.711	0.006	400	0	16,040	0
		30	0.700	0.003	400	0	16,040	0
**GAs**	**I**	80	1.038	0.004	600	0	24,040	0
		60	1.127	0.096	600	0	24,040	0
		30	1.044	0.012	600	0	24,040	0
	**II**	80	0.189	0.003	200	0	4020	0
		60	0.186	0.001	200	0	4020	0
		30	0.203	0.022	200	0	4020	0

## Conclusions and future recommendations

In this study a novel application of evolutionary heuristic paradigm based on Weighted Differential Evolution and Genetic Algorithms are exploited for accurate parameter estimation of nonlinear Hammerstein-Wiener systems with various noise scenarios and number of unknown elements in the parameter vector. Experimental results prove that both algorithms are reasonably convergent and accurate however the performance of WDE is relatively better by means of precision and complexity indices. Results through statistics validate that proposed evolutionary algorithms are quite efficient but performance of the algorithms declines as the noise level increases. Comparative analysis via different performance measuring indices i.e.*,* $\mathrm{MW}D_{hw\theta }$, $\mathrm{MA}E_{hw\theta }$, $\mathrm{RMS}E_{hw\theta }$, and $\mathrm{TI}C_{hw\theta }$ also validate the consistency of the designed procedures. Furthermore, computational complexity of GAs is found more than WDE based on time consumed, iterations completed, and function counts. Also, with the increase in the length of the parameter vector of the Hammerstein Wiener model, the complexity of WDE and GAs increases and same trend is observed with the increase in the noise levels. The novel designs evolutionary heuristics are indeed effective algorithms for parameter estimation problems of block-oriented models.

In future, the newly introduced nature inspired heuristics [54], [27], [55], [56], [50], [57] like firefly, gravitational search optimization algorithm, bat algorithm, ant bee colony optimization and their recently introduced fractional variants can be good alternatives to boost the accuracy of the proposed Hammerstein Wiener structures.

## Compliance with Ethics Requirements

***Human and animal rights statements*** *: All the authors of the manuscript declared that there is no research involving human participants and/or animal.*

***Informed consent*** *: All the authors of the manuscript declared that there is no material that required informed consent.*

***Data Availability:*** *My manuscript has no data associated with it.*

## CRediT authorship contribution statement

**Ammara Mehmood:** Conceptualization, Methodology, Software, Investigation. **Muhammad Asoif Zahoor Raja:** Visualization, Formal analysis, Validation.

## Declaration of Competing Interest

*The authors declare that they have no known competing financial interests or personal relationships that could have appeared to influence the work reported in this paper.*
